# The nonlinear trajectory of post-stroke aphasia recovery

**DOI:** 10.3389/fnhum.2026.1791894

**Published:** 2026-05-14

**Authors:** Jude O. Maraka, Yara Abukhaled, Sally M. Al Qaraghuli, Fatima A. Al Muhairi, Haia M. Abdulsamad, Hamdan Hamdan, Mohamed L. Seghier

**Affiliations:** 1 Department of Biological Sciences, College of Medicine and Health Sciences, Khalifa University, Abu Dhabi, United Arab Emirates; 2 Internal Medicine, The University of Tennessee Health and Science Center, Memphis, TN, United States; 3 Biomedical Engineering and Biotechnology Department, Khalifa University, Abu Dhabi, United Arab Emirates; 4 Research Institute for Health, Khalifa University, Abu Dhabi, United Arab Emirates

**Keywords:** brain reorganization, diaschisis, neuroplasticity, non-invasive brain stimulation, post-stroke aphasia, recovery, reperfusion

## Abstract

Post-stroke aphasia recovery is a dynamic process involving neural repair, compensatory reorganization, and behavioral optimization. While diaschisis, a functional disconnection of brain regions remote from the primary lesion, plays a pivotal role in the acute phase of recovery, its resolution and interaction with later stages remain underexplored. This narrative review synthesizes evidence from neuroimaging, neurophysiological, and clinical studies to integrate current understanding of post-stroke aphasia recovery mechanisms. Relevant literature was identified through targeted review of studies focusing on neuroplasticity and rehabilitation strategies. Building on this synthesis and on known existing models, we define a mechanistically grounded framework for aphasia rehabilitation, the Three-Tiered Recovery Model, to better describe the recovery trajectory. This model integrates emerging neurobiological insights with therapeutic timing to improve clinical outcomes. This framework integrates current evidence on neurovascular repair, diaschisis resolution, neuroplasticity, and therapeutic approaches, including vessel reperfusion, non-invasive brain stimulation, and behaviorally driven rehabilitation. Drawing on functional imaging and network-based models, we delineate three linked stages of recovery operating at distinct time scales: neural recovery (hours to days), compensatory reorganization (days to weeks), and behavioral optimization (weeks to months). Each tier is supported by distinct yet interacting mechanisms and intervention strategies. Early reperfusion and diaschisis resolution appear to set the stage for effective compensatory neuroplasticity and subsequent behavioral gains. Empirical evidence, including longitudinal and modeling-based studies, suggests that early interventions may exert disproportionately large effects on long-term outcomes, supporting a front-loaded recovery trajectory. Overall, by offering a revamped characterization of the well-established stroke framework, the Three-Tiered Recovery Model provides a clinically relevant framework for aligning intervention strategies with the biological stages of recovery, enabling a more individualized and mechanism-informed approach to post-stroke aphasia rehabilitation. Future work should validate this model through longitudinal imaging and precision rehabilitation trials.

## 1 Introduction

Post-stroke aphasia remains a significant neurological challenge, with recovery trajectories that are highly variable and difficult to predict. The prevalence of post-stroke aphasia is estimated to be 42% in acute care settings and up to 50% in rehabilitation settings (Flowers et al., 2016). However, due to heterogeneous evaluation methodologies, precise estimates remain elusive. While some patients regain language function over time, others experience only partial or no recovery (Dunn et al., 2016; Lazar and Antoniello, 2008). This heterogeneity complicates treatment selection, highlighting the need to understand the neural mechanisms underlying post-stroke recovery and brain reorganization to ultimately optimize and personalize treatment.

Research has increasingly focused on mapping post-stroke neuroplasticity to optimize rehabilitation strategies (Baron, 2025). Functional MRI (fMRI) is one of our most widely used tools for investigating brain reorganization and has demonstrated utility in both diagnostic and prognostic contexts (Saur et al., 2006). Additionally, neuroimaging biomarkers of structural integrity, such as fractional anisotropy derived from diffusion-weighted MRI, provide valuable predictors of recovery potential (Gaviria and Eltayeb Hamid, 2024). Measures such as lesion load and white matter integrity (white matter disconnection) have been shown to correlate with functional outcomes, supporting the development of more individualized rehabilitation approaches.

Traditionally, post-stroke recovery follows a three-stage trajectory: acute, subacute, and chronic phases. Although this classification provides a useful clinical structure, the underlying mechanisms of brain reorganization within and across these phases remain incompletely understood. Early recovery, typically occurring within the first 2 weeks, is characterized by initial language relearning (Koenig-Bruhin et al., 2013), whereas longer-term outcomes may extend over months to years, with only a subset of patients achieving full (Fridriksson and Hillis, 2021). For instance, Osa García et al. (2020) highlight that recovery is highly variable and influenced by multiple factors, including age (Ellis and Urban, 2016; Plowman et al., 2012), brain integrity (Kristinsson et al., 2022), initial aphasia severity (Lazar et al., 2010), treatment duration (Watila and Balarabe, 2015), educational background (Hillis and Tippett, 2014), premorbid language systems (Seghier and Price, 2018), and lesion size and location (Price et al., 2017). Importantly, the relative contribution of these factors varies across recovery, with early improvement more directly driven by neurobiological processes such as reperfusion and diaschisis resolution, whereas later recovery increasingly reflects the complex interaction between residual neural plasticity and behavioral or environmental factors.

Despite significant advances in understanding post-stroke aphasia recovery, important gaps remain in how these insights are translated into clinical practice. Existing frameworks often describe recovery in temporal terms (acute, subacute, chronic), yet these stages are not consistently linked to the underlying neurobiological processes that drive recovery. In parallel, mechanisms such as diaschisis resolution and interhemispheric reorganization have been extensively studied but are typically examined in isolation rather than integrated into a unified, stage-sensitive framework. As a result, there remains a disconnect between mechanistic insights derived from neuroimaging and experimental studies and their application to therapeutic decision-making. Addressing this gap requires an approach that aligns evolving biological processes with targeted intervention strategies across the continuum of recovery. A recent consensus study by an international multidisciplinary panel of stroke experts (Tiedt et al., 2026) has further highlighted that current clinical and imaging-based approaches lack sufficient biological precision to accurately predict stroke progression and recovery. More specifically, it highlighted the need for frameworks that incorporate dynamic, mechanism-based indicators of neural injury and repair, including emerging biomarker-driven approaches (Tiedt et al., 2026).

In this review, we examine the neurobiological mechanisms underlying post-stroke language recovery and emphasize the dynamic and stage-dependent nature of brain reorganization. Neural mechanisms of recovery in post-stroke aphasia refer to biological processes that restore, reorganize, or compensate for language function following brain injury. For instance, these include reperfusion of ischemic tissue, resolution of diaschisis, restoration of functional and structural connectivity, and neuroplastic changes within spared cortical and subcortical networks. Other factors such as age, education, co-morbidity, premorbid language systems, and treatment type and intensity are conceptualized as modifiers of recovery potential and can interact with neural processes in complex and nonlinear ways (Gerstenecker and Lazar, 2019). Here, we first outline the classical temporal phases of recovery to provide a structured clinical framework. We then synthesize the key mechanisms that characterize each phase, followed by a discussion of interhemispheric reorganization and therapeutic strategies. Finally, we introduce a mechanistically driven Three-Tiered Recovery Model that aligns intervention strategies with the dominant biological processes at each stage, offering a clinically relevant and individualized approach to post-stroke aphasia rehabilitation.

## 2 Evidence synthesis

This narrative review was conducted using a structured narrative approach to identify and synthesize evidence from neuroimaging, neurophysiological, and clinical studies relevant to post-stroke aphasia recovery. Relevant literature was identified through targeted searches of electronic databases, including PubMed, Scopus, and Google Scholar. Emphasis was placed on seminal prior work as well as recent publications addressing key mechanisms such as diaschisis, neuroplasticity, interhemispheric reorganization, and rehabilitation strategies.

Although the literature search was not systematic or comprehensive, we took a pragmatic approach that guided study selection by relevance to the biological processes underlying recovery and their implications for clinical intervention. Included sources comprised neuroimaging studies, longitudinal observational studies, and interventional trials examining therapeutic strategies across different stages of recovery. Due to the substantial heterogeneity of the stroke literature, findings were synthesized qualitatively, with attention to factors related to stroke stage, physiological factors, lesion characteristics, outcome measures, and therapeutic modalities.

To enhance conceptual rigor, the synthesis was informed by multiple complementary sources of evidence: (1) seminal highly-cited studies (e.g., Saur et al., 2006’s work is cited >1,000 times according to Web of Science); (2) critical appraisals by experts in the field (e.g., Baron (2025) offers a 5-decade critical summary of stroke pathophysiology, the work of Argye Hillis’ group in aphasia recovery, the work of Cathy Price’s group in degeneracy, among others), (3) recent consensus by experts in stroke research (e.g., the recent DELPHI-type consensus in Tiedt et al., 2026), and (4) existing meta-analyses that summarize consistent findings across neuroimaging studies (e.g., Stefaniak et al., 2021). Importantly, while we cannot rule out possible inherent bias in the evidence that emerged in this synthesis, our model (see below) remains coherent with existing stroke recovery frameworks.

In accordance with accepted principles for narrative reviews, emphasis was placed on integrating diverse evidence types and critically interpreting convergent findings. Formal evidence grading was not performed due to variability in study design, populations, and outcomes. Instead, the analysis focused on identifying consistent mechanistic and clinically relevant patterns to support a conceptually driven synthesis.

## 3 Classical phases of post-stroke aphasia recovery

A seminal fMRI study by Saur et al. (2006) delineated three distinct phases of aphasia recovery following left middle cerebral artery infarction. During the acute phase, fMRI revealed minimal activation of the left inferior frontal gyrus, correlating with severe speech disruption and low language recovery scores. 2 weeks later, significant upregulation was observed in the right inferior frontal gyrus, coinciding with early improvements in language function. In the chronic phase, peak activation shifted back to the left hemisphere, indicating functional normalization. These findings have been replicated in subsequent studies (Kiran, 2012; Koenig-Bruhin et al., 2013).

This commonly accepted three-phase framework depicts post-stroke recovery as a dynamic sequential process (Stefaniak et al., 2021): early repairs in the ipsilesional hemisphere, followed by contralesional compensatory activation, and ultimately, a re-shifting of function to the dominant (left) hemisphere. The extent of recovery is influenced by factors such as initial deficit severity (Lazar et al., 2010), lesion site, and preserved neural structures (Seghier and Price, 2023). For instance, Van Oers et al. (2018) and Wilson et al. (2022) emphasize that right-hemispheric compensation plays an important early role, but long-term recovery depends on left-hemispheric integrity. Wilson et al. (2022) further found that patients with milder deficits and preserved left-hemisphere structures exhibit faster and more robust recovery. Overall, prior work portrayed post-stroke recovery as a time-dependent interplay between intra- and inter-hemispheric activations and connectivity, with the ultimate goal to boost efficiency of spared processing pathways or recruit new pathways to compensate for the lost function. Some popular recovery models are discussed here; however, it is beyond the scope of this review to conduct a systematic comparison between all existing models of recovery. Instead, our aim is to sketch a simple model that captures different neuronal and behavioral facets of recovery after stroke.

Below, we discuss the most likely patterns of post-stroke brain reorganization across the acute, subacute, and chronic phases, with particular emphasis on the evolving interplay between neural disruption, compensatory recruitment, and long-term network reconfiguration. The three-phase model of recovery, as described by Saur et al. (2006), has been refined by many groups by further characterizing the underlying neurobiological mechanisms and approximate temporal boundaries of each stage. In particular, advances in functional and structural neuroimaging have enabled more precise mapping of phase-specific processes, including diaschisis and widespread network suppression in the acute phase, interhemispheric reorganization and contralesional recruitment during the subacute phase, and progressive normalization with experience-dependent plasticity in the chronic phase (Grefkes and Fink, 2011; Stefaniak et al., 2022; Seghier and Price, 2018). While these phases do not have rigid cutoffs and vary across individuals (Lazar and Antoniello, 2008), broadly defined timeframes derived from clinical and neuroimaging studies provide a useful structure for understanding recovery dynamics. These phases should therefore be interpreted as overlapping and dynamic, reflecting a continuum of recovery shaped by lesion characteristics, network integrity, and the timing and nature of therapeutic interventions (Wilson et al., 2022; Tilton-Bolowsky et al., 2024). Last but not least, it is also important to consider recovery as multi-factorial, reflecting the complex interplay between patient-specific and treatment-specific factors (Varkanitsa and Kiran, 2022; Bill et al., 2025).

### 3.1 Acute phase

The acute phase is characterized by a cascade of interrelated neuropathological processes that contribute to both local alterations and widespread network dysfunction. Reduced cerebral perfusion leads to depletion of ATP and loss of ionic homeostasis, resulting in neuronal depolarization and excessive glutamate release, which induces calcium influx and excitotoxic injury (Majumder, 2024). This is accompanied by oxidative stress, mitochondrial dysfunction, and early activation of inflammatory pathways, including microglial activation, cytokine release, and blood–brain barrier disruption (Kuriakose and Xiao, 2020). These processes extend beyond the infarct core into the surrounding penumbra, a region of metabolically compromised but potentially salvageable tissue, providing the biological substrate for early functional recovery with timely reperfusion (Dirnagl et al., 1999; Iadecola and Anrather, 2011; Campbell et al., 2019).

Regarding the neural mechanistic account of the acute phase, diaschisis plays a central role in language impairment. Diaschisis refers to dysfunction in structurally intact brain regions that are spatially distant from, yet functionally connected to, the primary lesion (Pasquini et al., 2022; Saur and Hartwigsen, 2012). Several forms of diaschisis have been described. Connectional diaschisis, which reflects alterations in structural and functional connectivity between distant brain regions, is thought to play a particularly important role in the early acute stage (Carrera and Tononi, 2014). Focal diaschisis refers to localized dysfunction in perilesional regions, often resulting from metabolic and vascular disturbances adjacent to the infarct, which could induce perilesional reorganization. Overall, diaschisis can be conceptualized as a network-level phenomenon involving functional and metabolic suppression across distributed but interconnected brain regions (Carrera and Tononi, 2014).

These disruptions arise because networks of conducting pathways link remote and peri-lesional regions to the primary lesion site. As a result, dysfunction in anatomically or functionally connected areas, whether adjacent or distant, can produce additional neurological deficits, consistent with the von Monakow doctrine of diaschisis (Vinychuk and Fartushna, 2021). Given its complexity, studying diaschisis after ischemic stroke requires diverse and innovative methodologies. Table 1 summarizes key approaches, their contributions, and associated limitations. Although several of these methodologies are primarily experimental or preclinical (Baron, 2025), they have been instrumental in shaping current understanding of diaschisis and network-level dysfunction, which in turn informs the interpretation of clinical neuroimaging findings and therapeutic strategies in post-stroke aphasia.

**Table 1:** Summary of key methodologies, their contribution to understanding diaschisis and their associated limitations.

Method	Type of model	Invasiveness	Key application	Advantages	Disadvantages
Vessel occlusion	Animal	Invasive	-Minimally ischemic stroke -Studies lesion propagation	-Reproduces real stroke physiology -Enables study of cortical & subcortical effects -Useful for testing therapies (Carmichael, 2005; Keister et al., 2024)	-Variable collateral circulation -Inconsistent lesion size/severity -Inflammation complicates diaschisis isolation (Mohajerani et al., 2011)
Clinical Inhibition	Human	Minimally invasive	-Pharmacologic suppression (GABAergic/ sodium channel blockers) -Stimulates functional disconnection	-Region-specific inhibition -Models diaschisis-like effects (Gold and Lauritzen, 2002)	-Less localized than vascular lesions -Variable duration of inhibition -Limited for long-term effects
Cortical Cooling	Animal/Experimental	Invasive	-Reversible deactivation of brain lesions -Short term functional studies	-Precise temporal control -Reversible model (Coomber et al., 2011)	-Poor spatial precision -Affects surrounding tissue -Limited targeting accuracy
Ontogenetics	Animal (Mainly)	Invasive	-Cell-type specific activation/inhibition -Long-range network modulation	-High spatial & temporal precision -Excitatory (ChR2) & inhibitory opsins -Network-level analysis (Alexander et al., 2009; Palmer et al., 2012; Cheng et al., 2014)	- Limited light diffusion -Uneven opsin expression -Requires advanced delivery systems -Thermal damage risk (Fenno et al., 2011)

Clinically, the acute phase, typically spanning the first hours to several days following stroke, is characterized by widespread network dysfunction and profound language impairment, often resembling global aphasia due to both local injury and remote suppression (Saur et al., 2006). Functional imaging studies (Stefaniak et al., 2021) demonstrate minimal activation in the left inferior frontal gyrus (IFG), correlating with severe language deficits and the lowest recovery scores (Saur et al., 2006; Stockert et al., 2020). The severity of these initial impairments (Lazar et al., 2010) is a key determinant of recovery potential, with milder deficits associated with more favorable outcomes (Saur et al., 2010). Early recovery during this phase is primarily driven by reperfusion, resolution of metabolic suppression, and restoration of functional connectivity within affected networks (Saur and Hartwigsen, 2012; Grefkes and Fink, 2011).

### 3.2 Subacute phase

The subacute phase, generally extending from days to weeks post-stroke, marks the beginning of functional recovery and may be further subdivided into an early subacute phase and a late subacute phase, reflecting distinct stages of recovery. This phase is characterized by dynamic reorganization of functional networks and progressive improvement in language function.

Early subacute recovery is largely attributed to the resolution of diaschisis and increased contralesional recruitment, particularly in patients with temporoparietal lesions, where reactivation of bilateral frontal cortices reflects recovery from diaschisis-induced dysfunction (Stockert et al., 2020). Neuroimaging studies demonstrate differential activation patterns linked to specific language components (Han et al., 2024). For example, fluency improvements at 2 weeks post-stroke correlate with activity in the right posterior supramarginal gyrus, insular cortex, and temporooccipital middle temporal gyrus. Between 2 weeks and 4 months, fluency enhancement is associated with activation in the left middle frontal gyrus, right temporooccipital middle temporal gyrus, and right middle frontal gyrus. Additionally, bilateral ventromedial prefrontal activation emerges as a predictor of recovery after controlling for baseline fluency (Stefaniak et al., 2022).

These findings are further supported by Wawrzyniak et al. (2022), who demonstrated that increased activation in high-lesion-connectivity regions correlates with improved language outcomes. This aligns with earlier reports from Saur et al. (2010), who identified bilateral frontal and left temporal activations within 2 weeks post-stroke. Together, these observations suggest that early right frontal cortex activation may serve as an important indicator of transition from the acute to the subacute phase. Accordingly, the subacute phase is widely recognized as a critical window of recovery, characterized by the resolution of diaschisis and increasing contralesional (right hemisphere) involvement (Tilton-Bolowsky et al., 2024), though right hemispheric involvement remains highly variable across patients and not always related to lesion factors (Turkeltaub et al., 2026). As recovery progresses into the late subacute stage, there is gradual re-engagement of ipsilesional networks and stabilization of emerging functional pathways, reflecting adaptive neuroplastic processes (Stefaniak et al., 2022).

At the biological level, the subacute phase represents a distinct and time-sensitive period of heightened neuroplasticity. Inflammatory cascades initiated during the acute phase continue to evolve, with damage-associated molecular patterns driving microglial activation, cytokine release, and leukocyte infiltration (Kuriakose and Xiao, 2020). Although these processes may contribute to secondary injury, they also create a permissive environment for structural remodeling. Concurrently, a transient “sensitive period” emerges, characterized by increased cortical excitability, altered excitation-inhibition balance, and reactivation of growth-associated gene expression patterns reminiscent of early neurodevelopment. These changes support synaptogenesis, dendritic spine formation, axonal sprouting, and large-scale network reorganization, particularly within peri-infarct regions and distributed functional networks.

Importantly, recovery during this phase reflects not only spontaneous biological repair but also the interaction between these processes and experience-dependent learning mechanisms. Behavioral training and rehabilitation can therefore shape and reinforce emerging neural pathways, contributing to the rapid gains typically observed during early recovery (Murphy and Corbett, 2009; Zeiler and Krakauer, 2013; Iadecola and Anrather, 2011; Krakauer, 2006).

### 3.3 Chronic phase

The chronic phase, typically beginning several months after a stroke, is characterized by functional normalization in fMRI activation and a re-shifting of peak activation back to the ipsilesional (left) hemisphere (Saur et al., 2010). This phase represents a major milestone in recovery, as most patients experience some degree of language restoration, though the level of restoration varies considerably across patients and time post-stroke.

Although aphasia severity often stabilizes around 6 months post-stroke, continued improvement or decline remains possible even years after the initial event (Ballester et al., 2019). Longitudinal studies highlight substantial variability in outcomes. For instance, a study of 39 patients with chronic left-hemispheric ischemic stroke found that 51% improved, 26% worsened, and 23% remained stable over time (Johnson et al., 2019). Similarly, Hope et al. (2017) demonstrated that while many patients reach an apparent recovery plateau, individual variability persists, with some continuing to improve or decline due to long-term neuroplasticity. Notably, these variations have been linked to structural adaptations in the right hemisphere, particularly in the anterior temporal lobe and premotor cortex.

Further evidence challenges the traditional plateau hypothesis. Smania et al. (2010) reported that significant language improvements can occur even decades after stroke, particularly in patients with large lesions. This suggests that chronic-phase recovery remains dynamic and is supported by ongoing neural plasticity while the brain’s atrophy is accelerating over time post-stroke (Naeser et al., 1998; Seghier et al., 2014).

At the network level, recovery in the chronic phase is increasingly supported by stabilization of functional systems and long-term reorganization of distributed language networks, with greater reliance on residual ipsilesional structures (Hamilton et al., 2011; Hope et al., 2017; Seghier and Price, 2018). Biologically, this stage is characterized by a shift from spontaneous repair processes toward experience-dependent plasticity and learning-driven reorganization. As the heightened plasticity of earlier stages diminishes, neural adaptation becomes more dependent on sustained behavioral training, guided by principles of use-dependent modulation, repetition, and task specificity. These processes promote synaptic strengthening, network refinement, and consolidation of newly established functional pathways across distributed cortical and subcortical systems.

Importantly, recovery during this phase often reflects a combination of functional restoration and compensatory strategies, both of which depend on learning mechanisms and may vary across individuals. While the rate of improvement typically slows, the persistence of plastic potential highlights the continued relevance of targeted rehabilitation in shaping long-term outcomes (Kleim and Jones, 2008; Cramer et al., 2011; Krakauer, 2006).

## 4 Ipsilesional versus contralesional post-stroke reorganization

Two primary mechanisms of neural recovery have been proposed following left-hemispheric stroke-induced aphasia: (a) restoration of damaged language regions within the ipsilesional left hemisphere, and (b) compensatory activation in the contralesional right hemisphere (Cao et al., 1999). While some patients exhibit concurrent recovery mechanisms, the exact role of interhemispheric interactions remains a subject of ongoing debate (Liu et al., 2024). For instance, some studies indicate that recovery strongly depends on connectivity within the left arcuate fasciculus, suggesting that therapy should focus on strengthening preserved neural networks, particularly in the ipsilesional hemisphere (Mansour, 2023). Moreover, Keser et al. (2020) investigated the compensatory role of the right hemisphere and found that excessive reliance on right-hemisphere structures, such as the arcuate fasciculus, can impair recovery, particularly in naming tasks. These findings suggest that while contralesional recruitment may facilitate early compensation, excessive reliance may be maladaptive and interfere with optimal functional restoration (Gajardo-Vidal et al., 2018).

On the other hand, Cocquyt et al. (2017) demonstrated that right-hemisphere activation is dynamic, peaking during early recovery and diminishing as language function shifts back toward the left hemisphere. This supports the notion that the right hemisphere plays a transient compensatory role, with long-term recovery more strongly dependent on ipsilesional restoration. Similarly, van Oers et al. (2018) reported that activation in the right posterior inferior temporal gyrus correlated with improved language outcomes, whereas excessive activation in the right inferior frontal gyrus was inversely associated with recovery. These findings highlight the region-specific and context-dependent nature of contralesional involvement in aphasia recovery (Stefaniak et al., 2021). Taken together, these observations suggest that although contralesional activation can support recovery, full functional restoration appears to depend on re-engagement of the ipsilesional (dominant) hemisphere. Future research should therefore focus on modulating interhemispheric interactions to optimize therapy-driven neural reorganization (Han et al., 2024).

An important consideration in translating these mechanisms into clinical decision-making is the relative contribution of lesion characteristics versus ongoing network-level physiology in shaping recovery trajectories and treatment response. While lesion location and volume remain critical determinants of initial deficit severity, growing evidence suggests that the current functional state of the language network, including patterns of diaschisis, connectivity disruption, and compensatory reorganization, may be more directly relevant for guiding therapeutic strategies (Seghier and Price, 2023). Recent advances in biomarker research further support this perspective, indicating that dynamic physiological states may provide a more accurate representation of ongoing injury and recovery processes than static structural measures alone. In this context, recent work by Billot and Kiran (2024) emphasizes the importance of disentangling distinct neuroplasticity mechanisms to better align intervention approaches with the underlying biological state of recovery. This perspective supports a shift from static lesion-based classification toward a dynamic, network-informed framework, in which treatment selection is guided by dominant recovery processes rather than anatomical injury alone (Billot and Kiran, 2024).

## 5 Therapeutic interventions in post-stroke aphasia

### 5.1 Early therapeutic interventions in the acute phase

The timing and type of intervention in the acute phase play a critical role in shaping long-term language recovery following stroke. Given that initial deficit severity is a strong predictor of recovery capacity (Lazar et al., 2010), early interventions aimed at optimizing neural reorganization are crucial. Evidence suggests that diaschisis resolution is central to early recovery, as functionally connected but structurally intact brain regions regain normal connectivity (Wawrzyniak et al., 2022). This raises an important clinical question: can targeted interventions accelerate this process to enhance language outcomes? In the following subsections, we discuss key therapeutic approaches, including reperfusion-based strategies and neuromodulatory interventions, that aim to facilitate early recovery.

### 5.2 Thrombolysis and endovascular reperfusion

Acute interventions such as intravenous thrombolysis and mechanical endovascular thrombectomy (MET) aim to restore cerebral perfusion, limit infarct expansion, and preserve language function (Meretoja et al., 2014). The success of these treatments is time-sensitive, with earlier intervention yielding superior functional outcomes (Meretoja et al., 2014). Hillis et al. (2006) demonstrated that reperfusion of the right temporo-occipital cortex significantly improved stimulus-centered neglect, underscoring the role of targeted blood flow restoration in language recovery. Similarly, perfusion of Broca’s and Wernicke’s areas has been associated with enhanced recovery potential, reinforcing the need for strategic revascularization (Hillis et al., 2006).

The shift from time-based to tissue-viability-based approaches has expanded therapeutic windows, allowing for thrombolysis up to 9 hours post-stroke (Demeestere et al., 2020; Chalet et al., 2022) and MET up to 16–24 h post-stroke, guided by DEFUSE-3 and DAWN criteria (Bücke et al., 2022; Mansour, 2023). These advancements facilitate the identification of salvageable brain tissue, making it potentially possible to treat patients with unknown stroke onset, such as wake-up strokes, thereby improving patient selection and outcomes.

### 5.3 Neuromodulation and emerging therapies

Among these approaches, neuromodulatory strategies have emerged as promising tools for directly influencing network-level plasticity (Xia et al., 2025). Beyond reperfusion, neuromodulatory interventions such as optogenetics and non-invasive brain stimulation are being explored to enhance neural plasticity. Optogenetic stimulation of perilesional neurons has shown promise in accelerating cortical reorganization (Fenno et al., 2011; Cheng et al., 2014). Similarly, transcranial magnetic stimulation (TMS) and transcranial direct current stimulation (tDCS) have been used to modulate cortical excitability, either by enhancing ipsilesional activity or suppressing maladaptive contralesional activation (Naeser et al., 1998; Hamilton et al., 2011).

Specifically, non-invasive brain stimulation techniques, such as repetitive TMS and tDCS, can enhance neuroplasticity (Williams et al., 2024; Gholami et al., 2022) and are primarily utilized during the subacute phase of recovery where compensatory reorganization becomes the focus. Repetitive TMS has been shown to modulate interhemispheric imbalances by inhibiting overactivity in the contralesional hemisphere (e.g., right Broca’s homolog). For instance, targeting the right inferior frontal gyrus with repetitive TMS has demonstrated positive effects on naming, repetition, and overall language function (Xie et al., 2025), with some studies reporting long-lasting benefits up to 12 months post-treatment (Gholami et al., 2022; Harvey and Hamilton, 2022).

Similarly, tDCS offers flexibility by using anodal stimulation to excite perilesional regions or cathodal stimulation to inhibit maladaptive activity in the contralesional hemisphere (Marangolo, 2020; ALHarbi et al., 2017). This modality has shown efficacy in improving naming abilities and other language domains (Wang et al., 2026), although variability in stimulation parameters, participant heterogeneity, and methodological limitations remain challenging (ALHarbi et al., 2017; Schlaug et al., 2011). Combining brain stimulation with behavioral therapies, such as speech-language therapy, has shown potential for synergistic effects, particularly when stimulation is individualized based on lesion characteristics and guided by neuroimaging (Arheix-Parras et al., 2021; Williams et al., 2024).

Despite its potential, the field of brain stimulation for post-stroke aphasia is still evolving, with many questions remaining unanswered. The mechanisms underlying its effects on language recovery are complex, involving both ipsilesional and contralesional hemispheres in a dynamic interplay that changes over time (Schlaug et al., 2011). Early post-stroke, the contralesional hemisphere often compensates for language deficits, but excessive reliance on this hemisphere may lead to inefficient recovery, emphasizing the importance of later re-shifting functions to the ipsilesional hemisphere (Schlaug et al., 2011; Arheix-Parras et al., 2021). Neuroimaging studies have begun to clarify these mechanisms, suggesting that repetitive TMS and tDCS influence not only local cortical excitability but also network-wide plasticity, including modulation of remote perilesional and homologous contralesional areas (Hara and Abo, 2021; Shah et al., 2013).

Additionally, newer approaches, such as high-definition tDCS and multimodal interventions combining brain stimulation with neuroimaging techniques like fMRI, offer opportunities for more precise and individualized treatments (Arheix-Parras et al., 2021; Shah et al., 2013). However, standardized protocols, larger randomized controlled trials, and long-term follow-ups are needed to establish the clinical efficacy and safety of brain stimulation, as well as its integration into routine rehabilitation programs (Williams et al., 2024; Marangolo, 2020; ALHarbi et al., 2017). Moving forward, refining these techniques to consider patient-specific factors such as lesion site and size, aphasia severity, and cognitive reserve will be essential to optimize outcomes and exploit the full potential of brain stimulation in post-stroke aphasia rehabilitation (Arheix-Parras et al., 2021; Harvey and Hamilton, 2022).

Overall, as aphasia recovery varies considerably across patients, neuroimaging-guided rehabilitation strategies are critical for optimizing personalized interventions. Future research should integrate acute reperfusion strategies, neuromodulatory therapies, and individualized rehabilitation to refine precision medicine approaches and maximize post-stroke language recovery.

## 6 A three-tiered recovery model in post-stroke aphasia

While existing models have advanced our understanding of post-stroke language recovery, a key challenge remains: translating complex neurobiological mechanisms into stage-sensitive and clinically actionable therapeutic strategies. Aphasia recovery is a dynamic and nonlinear process shaped by a complex interplay of structural and functional changes, compensatory neuroplasticity, and behavioral adaptation. Numerous frameworks have been proposed to explain this process, including dual-stream models of language processing, the interactive two-process model, and network-based theories emphasizing degeneracy and functional reallocation (Stefaniak et al., 2020). These frameworks have advanced our theoretical understanding of language reorganization, yet a persistent challenge remains: translating this complexity into stage-sensitive and personalized therapeutic strategies. For example, the interactive two-process model distinguishes between automatic and effortful processing in language recovery, while network models such as those proposed by Price and Friston (2002) highlight degeneracy as key to explaining and predicting recovery at the individual patient level (Price and Friston, 2002; Seghier and Price, 2018; Dai et al., 2024).

**Figure 1:**
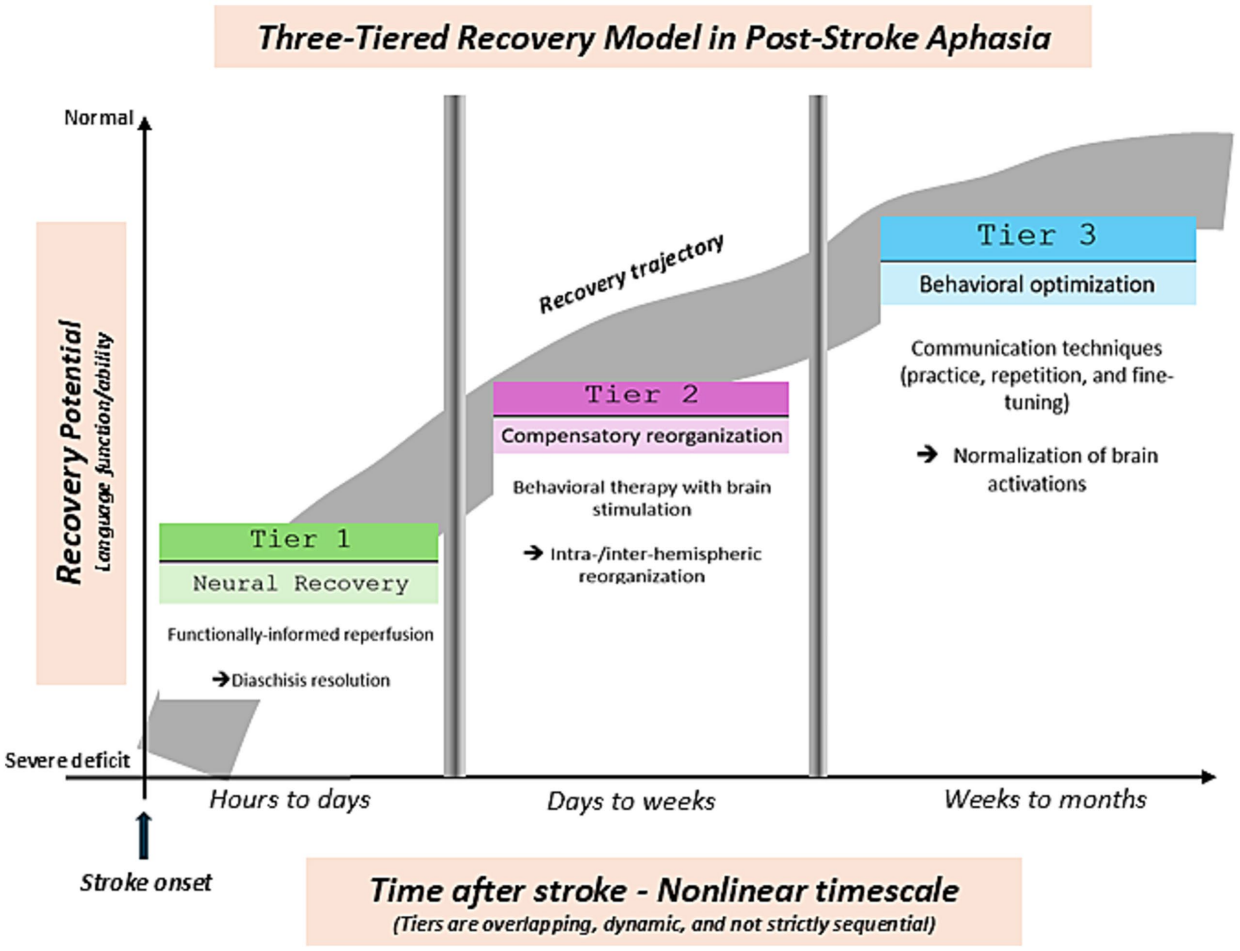
Schematic representation of the Three-Tiered Recovery Model illustrating the nonlinear and dynamic progression of language recovery following stroke. The Y-axis represents recovery potential (operationalized as overall language function or ability as assessed with clinical tests), ranging from severe language deficits to near-normal function, while the (nonlinear) X-axis represents time post-stroke. The temporal ranges (~hours to days, ~days to weeks, ~weeks to months) reflect a nonlinear timescale across phases/tiers but are approximate and intended to illustrate general trends rather than fixed or discrete phases (cut-offs between phases/tiers are shown as vertical bars with gradients to illustrate variable transitions across patients). The rising curve represents a nonlinear recovery trajectory, reflecting variable rates of improvement over time, and might include periods of rapid gain, plateau, or slower progression. The curve is drawn at high thickness to reflect uncertainty and variability across time and patients. Tiers are overlapping and not strictly sequential, and individual recovery trajectories may vary based on lesion characteristics, network integrity, and therapeutic interventions. T*ier 1 (Neural Recovery; ~hours to days):* Early phase characterized by diaschisis resolution and functionally informed reperfusion aimed at restoring neural connectivity. *Tier 2 (Compensatory Reorganization; ~days to weeks):* Intermediate phase involving recruitment of alternative neural pathways, including intra- and inter-hemispheric reorganization, often supported by behavioral therapy and neuromodulatory interventions. *Tier 3 (Behavioral Optimization; ~weeks to months):* Later phase focused on consolidation and refinement of function through communication techniques (practice, repetition, and fine-tuning), alongside progressive normalization of brain activation patterns.

In this context, we propose the Three-Tiered Recovery Model as a refined characterization of the well-established multi-phase recovery framework in stroke research (e.g., Saur et al., 2006). The model is a clinically oriented conceptual framework that helps to operationalize complex recovery phases into explainable and treatment-oriented phases. The model is not intended to replace existing models and frameworks but rather to serve as a heuristic “scaffold” that aligns therapeutic interventions with predominant neurobiological processes across recovery. By emphasizing a mechanism-driven and outcome-informed rather than purely time-based approach, the model invites clinicians to tailor rehabilitation strategies based on evolving patterns of neurovascular repair, interhemispheric compensation, and behaviorally reinforced plasticity (Figure 1).

To clarify its contribution relative to existing frameworks, the Three-Tiered Recovery Model both builds upon and extends prior models of aphasia recovery. It retains key insights from established perspectives, including distributed language networks (dual-stream models), the distinction between automatic and effortful processing (interactive two-process model), and the concept of degeneracy and functional reallocation in network-based frameworks. However, unlike these primarily descriptive or mechanistic models, the present framework organizes these processes into phase-sensitive dynamics aligned with recovery progression and explicitly links them to therapeutic timing and intervention strategies. The model tentatively offers a translational bridge between mechanistic understanding and clinical decision-making.

The Three-Tiered Recovery Model was developed through an integrative synthesis of findings from neuroimaging, neurophysiological, and clinical rehabilitation studies. Recurring patterns across longitudinal imaging studies, mechanistic investigations of diaschisis and neuroplasticity, and interventional research were analyzed to identify common trajectories of recovery. These patterns were then conceptually grouped into stages defined by dominant biological processes rather than fixed temporal boundaries. The model therefore represents a synthesis of converging evidence rather than a reinterpretation of existing temporal classifications, offering a clinically meaningful structure for aligning intervention strategies with the evolving neurobiological state of recovery.

Importantly, the Three-Tiered Recovery Model is not intended to classify patients solely based on time since stroke, but rather according to the predominant biological and functional processes shaping recovery at a given stage. In clinical practice, phase recognition may be informed by a combination of factors, including initial aphasia severity, lesion size and location, preservation of structural connectivity, neuroimaging findings, and response to therapy. For example, patients in whom early recovery is dominated by reperfusion dynamics, diaschisis resolution, and rapidly evolving neurological deficits may be understood as operating within Tier 1 processes. In contrast, the emergence of contralesional recruitment, interhemispheric rebalancing, and adaptive engagement of alternative pathways may reflect Tier 2 predominance. In later stages, when deficits become more stable and recovery is increasingly shaped by experience-dependent plasticity, behavioral adaptation, and therapy-driven consolidation, Tier 3 processes may be most relevant. Put another way, the exact timing of the different phases varies across patients as well as with recovery outcome.

Practically, application of the Three-Tiered Recovery Model involves identifying the dominant biological and functional processes shaping recovery, rather than relying on elapsed time alone. In clinical practice, this can be guided by a combination of neurological trajectory, neuroimaging findings, and response to early interventions. In this context, emerging biomarker-based approaches may provide a critical means of operationalizing recovery stages by capturing dynamic pathophysiological processes, including neuronal injury, inflammation, and neuroplasticity. Such markers have the potential to complement neuroimaging and clinical assessment, enabling more precise alignment between the biological state of recovery and therapeutic decision-making (Tiedt et al., 2026). For instance, patients demonstrating rapid neurological change, fluctuating deficits, or evidence of salvageable tissue on perfusion imaging are likely operating within Tier 1 (neural recovery), where reperfusion and stabilization of the neural environment should be prioritized. In contrast, the emergence of contralesional activation, interhemispheric imbalance, or early functional gains despite persistent deficits may indicate a transition to Tier 2 (compensatory reorganization), where interventions such as neuromodulation and targeted behavioral therapy can facilitate adaptive network recruitment. In later stages, when deficits become more stable and recovery is increasingly shaped by experience-dependent learning and behavioral adaptation, patients may be understood as operating within Tier 3 (behavioral optimization), where structured, task-specific care is most effective.

Importantly, these phases are not mutually exclusive, and patients may exhibit overlapping features across phases. This framework therefore supports a dynamic, process-based approach to treatment selection, allowing clinicians to tailor interventions according to the evolving neurobiological state of recovery rather than fixed temporal cut-offs. The strength of evidence underlying these domains varies substantially, ranging from high-confidence clinical interventions (e.g., IVT/MT) to mechanistic inferences (e.g., diaschisis) and emerging interventional approaches (e.g., neuromodulation), and should be interpreted within this spectrum.

### 6.1 Tier 1: Neural recovery (timescale: hours to days)

Neural recovery refers to the brain’s ability to repair and reorganize itself after a stroke. In the early hours to days immediately after the stroke, the focus must be on the reperfusion of damaged areas, restoring blood flow to ischemic tissue. This phase is critical, as it initiates the healing process and begins the resolution of edema. Early intervention during this phase is essential to support recovery, and neuroprotective strategies may help to limit further neuronal damage. Importantly, recovery potential during Tier 1 may vary depending on stroke characteristics. In a study of patients with pure aphasia, those with small infarct volumes and low NIHSS scores showed rapid symptom resolution in the subacute phase, while persistent aphasia was associated with cardioembolic or atherosclerotic stroke types and extensive frontal lobe damage. This suggests that early neural recovery may depend not only on timely reperfusion but also on stroke etiology and the integrity of domain-general cognitive networks supporting language (Bill et al., 2025).

As an optimal treatment strategy, we recommend reperfusion strategies, such as thrombolysis (if within the treatment window), and early rehabilitation efforts to prevent complications like muscle atrophy and joint contractures. In addition to reperfusion strategies, one can also mention drug-based treatments. For instance, Cerebrolysin has shown promise as a pharmacological adjunct in early stroke recovery. Acting through neuroprotective and neurotrophic mechanisms, it may help stabilize the neural environment, reduce secondary damage, and support early reorganization (Homberg et al., 2025). In the ESCAS trial, patients who received Cerebrolysin within 3–5 days post-stroke showed significantly greater improvements in language and neurological function compared to placebo. These findings suggest that Cerebrolysin may aid diaschisis resolution and enhance early recovery, making it a valuable addition to Tier 1: Neural Recovery.

Evidence supporting Tier 1 processes is strongest for reperfusion-based interventions (IVT/MT), which are supported by robust clinical data, whereas supporting neuroimaging findings (e.g., diaschisis resolution) and pharmacological treatments provide complementary but more variable mechanistic and clinical evidence.

### 6.2 Tier 2: Compensatory reorganization (timescale: days to weeks)

When full recovery of the original neural pathways is not possible, the brain engages in compensatory reorganization. In this phase (days to weeks post-stroke), alternative brain regions take over the functions of the damaged areas. For example, if the left (dominant) hemisphere is damaged, the right hemisphere may begin to support language recovery through adaptive plasticity. This phase leverages the brain’s ability to recruit spared contralateral or ipsilateral regions to restore function.

Recent resting-state fMRI studies have identified distinct patterns of neural compensation depending on lesion severity and structural integrity (Feng et al., 2025). Bilateral or predominantly ipsilateral recruitment of language regions has been linked to recovery in naming and spontaneous speech, while contralesional homologous activation supports comprehension and recognition tasks. In more severe cases, non-language brain regions such as the sensorimotor and visual cortices may also be recruited to aid fluency recovery. These findings reinforce the idea that adaptive plasticity is not uniform, and effective interventions must be tailored to each patient’s connectivity profile (Homberg et al., 2025).

Therefore, the role of any intervention during this stage should be to facilitate the recruitment of spared brain pathways capable of supporting impaired functions. As an optimal strategy, we recommend combining behavioral therapy with brain stimulation techniques such as tDCS and TMS, both of which can modulate cortical excitability and enhance functional reorganization. Brain stimulation can target spared regions that can sustain recovery, as evidenced by functional neuroimaging studies. For instance, if a task can be sustained by either pathway X or Y, then brain stimulation can target pathway Y in patients with damage to pathway X (and vice versa). When guided by neuroimaging, these tools can help clinicians promote targeted recovery pathways rather than relying on spontaneous adaptation alone.

Evidence for Tier 2 processes includes functional neuroimaging studies and interventional trials of neuromodulation (e.g., tDCS, TMS), with heterogeneous and context-dependent outcomes that reflect evolving and variable levels of clinical evidence (Brady et al., 2025).

### 6.3 Tier 3: Behavioral optimization (timescale: weeks to months)

Weeks or months after stroke, behavioral optimization becomes vital. Even if the brain has successfully compensated for lost functions, patients often require structured therapy to consolidate and refine newly formed pathways. Notably, emerging evidence suggests that recovery may continue even without formal intervention during this chronic phase. Anthony et al. (2025) demonstrated significant improvements in aphasia severity over time in individuals with chronic stroke, even in the absence of speech therapy, underscoring the brain’s inherent capacity for delayed spontaneous recovery (Anthony et al., 2025).

However, targeted behavioral therapies, such as constraint-induced language therapy, can build upon this natural recovery, helping patients fine-tune compensatory strategies and optimize communicative effectiveness. When informed by neuroimaging and tailored to individual needs, these interventions can amplify and stabilize long-term gains. Overall, a positive and stimulating environment (Okwaraji et al., 2026), whether enabled by healthcare professionals or family members, is essential to improve and maintain recovery even years after stroke.

Evidence for Tier 3 processes is supported by rehabilitation studies emphasizing experience-dependent plasticity and therapy intensity, although outcomes vary across patient populations, intervention designs, and long-term follow-up measures (Brady et al., 2025).

### 6.4 Bridging the gap between pathophysiology and rehabilitation planning

Traditional frameworks often categorize post-stroke aphasia recovery into acute, subacute, and chronic phases based on elapsed time. While clinically useful, these time-based classifications may overlook the underlying neurobiological processes that actually shape recovery. The Three-Tiered Recovery Model proposed here reframes recovery as a mechanistically driven continuum, wherein rehabilitation strategies are aligned with evolving patterns of neural repair, plasticity, and behavioral adaptation, regardless of strict temporal boundaries.

Unlike models that treat phenomena such as diaschisis resolution, compensatory reorganization, and behavioral therapy as discrete or sequential, our framework integrates them into a continuous and overlapping process. It acknowledges that these mechanisms often co-occur and influence one another in a nonlinear fashion. Importantly, the model does not suggest a fixed recovery trajectory, nor does it discount the potential for meaningful gains in the chronic phase. Instead, it highlights that early interventions, such as reperfusion and targeted neuromodulation, can set the stage for more effective long-term outcomes, especially when tailored to individual neuroanatomical profiles.

Beyond lesion-based models, emerging evidence suggests that aphasia may represent a breakdown in distributed systems integration, not just focal language loss, thus inviting clinicians to integrate a network-level understanding of aphasia when designing therapies. In this context, functional recovery reflects the brain’s capacity to re-coordinate activity across multiple networks and domains, including language, executive, attentional, and sensorimotor domains (Rangus et al., 2026; Xu et al., 2026). The Three-Tiered Recovery Model accommodates this system’s perspective by aligning phase-specific interventions with the evolving demands of network-level reintegration, rather than isolated linguistic repair.

Empirical studies further highlight the importance of early intervention, while also underscoring ongoing questions regarding the optimal alignment between intervention type and recovery phase. For example, Godecke et al. (2013) demonstrated that the amount of aphasia therapy provided in the first month post-stroke significantly predicted long-term communication outcomes (Godecke et al., 2013). However, this behavioral approach in the acute phase raises an important consideration, as it may not fully align with strictly phase-specific, mechanism-driven intervention models. Instead, it suggests that early behavioral engagement may still be beneficial even outside traditionally defined mechanistic windows. A systematic review by Husak et al. (2023) further reinforced that early therapy timing, not just intensity, is associated with more favorable language and communication outcomes (Husak et al., 2023). Together, these findings support a more flexible and integrative view of recovery, in which intervention’s timing and modality may overlap across phases.

Finally, by emphasizing a nonlinear (e.g., logarithmic) recovery trajectory, the model offers a way to conceptualize why early therapeutic efforts often produce outsized long-term benefits. More importantly, it provides a clinically actionable framework for aligning neuroimaging, brain stimulation, and behavioral therapies with the dynamic stages of recovery. Unlike existing time-based models, our model incorporates recent findings from neuroimaging, pharmacologic intervention, and longitudinal outcome studies to support a more biologically grounded and clinically actionable staging system. Rather than replacing existing models, our Three-Tiered Model builds upon them to bridge the gap between pathophysiological insight and real-world rehabilitation planning.

### 6.5 Translational considerations and limitations

Post-stroke recovery remains a complex phenomenon influenced by multiple interacting factors, including spontaneous biological recovery, behavioral compensation, and variability in therapy intensity and timing. As such, the relationships described in this model should be interpreted as integrative rather than strictly causal (Seghier and Price, 2023). Additionally, the strength of evidence supporting different components of the model varies (Tiedt et al., 2026), ranging from well-established clinical interventions (e.g., reperfusion therapies) to emerging or exploratory approaches (e.g., neuromodulation and preclinical techniques). These differences should be considered when applying the model in clinical settings.

## 7 Conclusion

The trajectory of language recovery after stroke is complex, dynamic, and highly variable, shaped by an interplay of neurovascular repair, structural plasticity, and functional reorganization. While traditional time-based frameworks offer a general structure for staging recovery, they do not fully account for the evolving neural mechanisms that drive rehabilitation success. In this review, we introduced the Three-Tiered Recovery Model as a flexible, mechanistically grounded framework to help align interventions with biological processes active at different recovery phases.

*Tier 1* (Neural Recovery) emphasizes the importance of early reperfusion and the resolution of diaschisis, setting the foundation for neuroplastic adaptation. *Tier 2* (Compensatory Reorganization) captures the brain’s capacity to recruit spared networks and adapt functionally, often guided by targeted stimulation and behaviorally relevant tasks. *Tier 3* (Behavioral Optimization) underscores the role of long-term therapy in consolidating gains and refining communicative behavior through structured repetition and practice. These stages are not strictly sequential and may overlap or evolve nonlinearly, depending on lesion characteristics and patient-specific factors (Tilton-Bolowsky et al., 2024).

Rather than offering a definitive path, the Three-Tiered Recovery Model is intended as a hypothesis-generating, conceptually driven framework that integrates current evidence under a mechanistic lens and supports a more targeted, dynamic understanding of aphasia rehabilitation. The model also accommodates emerging pharmacological strategies that align with and support phase-specific recovery mechanisms. Moving forward, larger longitudinal studies of individuals (Hillis, 2025), integrating neuroimaging, neuromodulation, and behavioral outcomes, will be essential to validate recovery trajectories and optimize intervention timing. Identifying biomarkers that predict responsiveness to therapy, particularly at the individual patient level, will further strengthen our ability to deliver precision rehabilitation for aphasic patients.
